# The Psychology of Charities

**Published:** 1917-02

**Authors:** T. D. Crothers

**Affiliations:** Hartford, Conn.


### The Psychology of Charities.

BY T. D. CROTHERS, M. D., HARTFORD, CONN.

The average business man devotes a large part of his time to the accumulation of property. Every success intensifies the desire to accumulate more, and very often this is followed by mistakes in judgment and losses, and renewed efforts to recover constitute a large part of his everyday work. Everything else is subordinated to this one great object. Even when his accumulations exceed every possible want or necessity, even up to the stage of luxury the desire to continue increases and becomes a vivid mania that continues through life. When the shadows deepen and the prospect of death looms up, then the question of how to dispose of it must be answered. Advisers and lawyers suggest, but somehow he is distracted and helpless. The contested wills and the settlement of estates show the imbecility of the testator. While he was able to gather the property with some skill and good judgment, he had the most infantile conceptions of how the property should be disposed of. Leaving it all to his family meant their ultimate ruin. Leaving it to the church on the supposition that it would in some way atone for his failure to exercise his spiritual privileges is full of conflicting doubts and the disappearance of the property with little or no tangible records of its destiny.

This is the history of the average business man who in a large proportion of cases dies with a very small amount of property to his credit. A number of such persons have at some time or another been in possession of very large amounts, the loss of which has been very injurious to their health and ambition. Some of them never recover a loss of this kind. Others become more energetic and determined, even after repeated losses.

A certain number of business men recognize the necessity of placing their capital in some way so that it will do the largest amount of good in the future. They write wills and consider the question of endowments and what could be done that will promise the most and best returns for what they call their hard-earned capital.

A very curious history could be made up from this point of view. In fact, every town and city in the country where men have accumulated large amounts of property afford startling illustrations of the incapacity and foolishness of persons to wisely place their surplus means for the benefit of the world. Among the numerous examples is this one:

A man whose son died in college left an enormous sum as a memorial, and this was expended in a palatial building of the most elaborate character, and its gift to the college added a burden to keep it up.

Another man gave a magnificent memorial church to a little inland village who could hardly support a church of any kind before. A hospital, an old people's home, a chapel and so on through a long list of buildings as charities given here and there with little or no reference to the surroundings and practical character of the work they are intended to do are sadly misplaced dispositions of property. Thus one hospital had five free beds, and the managers were obliged to charge the occupants of these rooms whenever it was possible, simply to pay expenses. Two professors' chairs in a college were endowed, but the occupants were urged to scale down the salaries to help the other departments of the college. A free church with the minister's salary paid from funds was practically abandoned from apathy, indifference in the minister and trustees. Money sent abroad for schools in missionary fields is subject to such a heavy drain of expenses that it often fails to accomplish the good intended. These are all charities good in themselves, but the methods of applying them in actual activities of life are faulty. This is the shady side of the subject. Reverse this and some very striking examples of grand helpful charities can be found. Take

Gerard College as one instance. Here a free school and college for indigent and homeless boys has been going on for years doing grand work in the most practical realistic ways. Johns Hopkins' hospital, medical school and research work is another charity where researches in science and methods of healing can be carried on in the most satisfactory way, and its blessings extend to every possible direction. These are two of the most prominent examples of a large number of smaller institutions.

A Chicago physician who had acquired a large property from the rise of real estate decided to give away his property personally, and not leave it to lawyers and others, or to the faulty construction of some will. Among one of his charities was this:

A Southern college appealed to him for a hospital building to accommodate sick students. On examination he decided that they needed a new water supply and not a hospital, so he bought land and built a modern waterworks for the conveying of water to the college, and this settled the problem. In another institution he found unsuitable buildings to accommodate poor boys and girls who could not afford to pay room rent. He built a plain dormitory and left it endowed to supply this want. To his own family he set aside certain sums of money in trust, requiring release papers for any obligations of the future. Finally the doctor died, and there were no legal questions or claims or lawyers to help divide the property. In this he left a grand object lesson to business men and others, not to leave property for others to divide, but to settle it during life and attend to its disposition himself.

Another object lesson of how money can be placed to do the largest possible good to the world was that of a man dying in St. Bartholomew's Hospital in London over a century ago. He had made a great fortune abroad, and was taken sick and went to the hospital. Realizing that he could not live, he took the advice of a physician and left all his property to a school for homeless boys. This has been going on for over a century and is one of the great charities of London, taking up boys lost and abandoned on the streets, training and educating them until fifteen years of age and over, then sending them out into the world. What a grand consummation this is to a life of strenuous effort in the accumulation of money. In the world of science there are evidences in the different laboratories of the value of endowments, which will enable trained observers to take up disputed questions of health and disease and determine the laws which control them, thus actually adding untold wealth in diminished disease and increased longevity.

The laboratory work on the alcoholic problem through the means of endowed funds has revolutionized much of the present theories of the past and revealed a new world of facts that no speculation of opinions could ever determine. The great scourges of the human race have practically disappeared through the march of scientific studies and more accurate examinations of the facts. Here is a limitless field for doing good and lifting the race up higher and making life broader and richer in health and happiness.

We are confronted today with one of the greatest questions of civilization, viz.: How to perpetuate life, diminish its disease and sorrow, increase the happiness and satisfaction of living.

Alcohol is one of the most serious perils that must be overcome before this can be attained. An army of enthusiastic laymen are pressing different solutions and measures for relief, and a most conflicting literature and agitation are growing out of this subject. On the other hand, a small body of scientists are trying to determine the causes and conditions which are at the bottom of this great evil. When these are discovered, the measures for relief can be applied which will literally stamp out this evil as fully as typhoid and yellow fever has been obliterated. Work of this kind must depend on endowments because of its magnitude, requiring highly trained workers who give their entire time to the subject. There is a psychology to this which appeals to every medical man because of its immediate and intense personal benefits. Physicians can influence capitalists and show how this new field of charity can be put to personal service.

A research hospital has been established at Hartford, Conn., to take up the causes of alcoholism and inebriety, and explore an entirely new country that is unknown up to the present. The laboratories have shown us what alcohol is on cell and tissue and that is the beginning, but what causes, precedes and favor the drink craze are questions to be solved in the future. Here is a veritable charity where money can be put to service, bringing far more practical returns in an improved race than in any commercial ventures. There is a psychology along this line that deserves the warmest consideration and attention, and that is this:

How to put capital into service to develop and widen human activity and lessen human suffering and pain. Every physician is doing this in a practical way in the cup of cold water, in the kindly advice and the innumerable attentions and activities and the suffering poor who can give no return except thanks.

The charity of today means something personal, vital and real and the Hartford foundation is based on this fact. We must know what this problem is before we can apply proper preventive measures, and we must appeal for help to make such observations, not in the low sense of the word—charity—but in the broad conception of measures that will help evolve and develop humanity to a higher plain of living. This is the new field of practical psychology and practical charity that calls for recognition in these times of stress and trial.

